# Supplementary material for: Cell-permeable succinate prodrugs rescue mitochondrial respiration in cellular models of acute acetaminophen overdose
Source: PLoS One. 2020 Apr 6;15(4):e0231173. doi: 10.1371/journal.pone.0231173 (PMC7135280; doi:10.1371/journal.pone.0231173)
Supplement: Attachment — Submitted filename: Response to Reviewers.docx [file pone.0231173.s002.docx]

Dear editor,

thank you for inviting us to resubmit our manuscript (PONE-D-20-01070). We are grateful for the constructive comments from the editor and the reviewers. We have carefully addressed all their concerns. The editors and reviewers’ comments are displayed below in normal font style and the specific response to each issue raised in the comments is displayed italic font style.

For the authors,

Sarah Piel

Journal Requirements:

When submitting your revision, we need you to address these additional requirements.

1. Please ensure that your manuscript meets PLOS ONE's style requirements, including those for file naming. The PLOS ONE style templates can be found at

http://www.journals.plos.org/plosone/s/file?id=wjVg/PLOSOne_formatting_sample_main_body.pdf and <http://www.journals.plos.org/plosone/s/file?id=ba62/PLOSOne_formatting_sample_title_authors_affiliations.pdf>

*Answer: The manuscript and figures have now been updated to meet PLOS ONE’s style requirements.*

2. Please note that all PLOS journals ask authors to adhere to our policies for sharing of data and materials: https://journals.plos.org/plosone/s/data-availability. According to PLOS ONE’s Data Availability policy, we require that the minimal dataset underlying results reported in the submission must be made immediately and freely available at the time of publication. As such, please remove any instances of 'unpublished data' or 'data not shown' in your manuscript and replace these with either the relevant data (in the form of additional figures, tables or descriptive text, as appropriate), a citation to where the data can be found, or remove altogether any statements supported by data not presented in the manuscript.

*Answer: Any instances of ‘unpublished data’ have now been replaced with a reference to additional supplementary figures.*

3. Thank you for stating the following in the Financial Disclosure section:

'E.E.: Swedish government project and salary funding for clinically oriented medical research (ALF-grants; F 2014/354) URL: https://www.skane.se/en/politics-and-organisation/research/services-and-support-for-researchers/grants-and-applications/

E.E.: Regional research and development grants (Southern healthcare region, Sweden; 170083): URL: https://www.skane.se/en/politics-and-organisation/research/services-and-support-for-researchers/grants-and-applications/

E.E.:The Crafoord Foundation (2017-0776) URL: https://en.crafoord.se/

M.J.H.:The Royal Physiographic Society in Lund (20141112) URL: https://www.fysiografen.se/en/

The funders had no role in study design, data collection and analysis, decision to publish, or preparation of the manuscript.'

We note that one or more of the authors are employed by a commercial company: NeuroVive Pharmaceutical AB

a. Please provide an amended Funding Statement declaring this commercial affiliation, as well as a statement regarding the Role of Funders in your study. If the funding organization did not play a role in the study design, data collection and analysis, decision to publish, or preparation of the manuscript and only provided financial support in the form of authors' salaries and/or research materials, please review your statements relating to the author contributions, and ensure you have specifically and accurately indicated the role(s) that these authors had in your study. You can update author roles in the Author Contributions section of the online submission form.

Please also include the following statement within your amended Funding Statement.

“The funder provided support in the form of salaries for authors [insert relevant initials], but did not have any additional role in the study design, data collection and analysis, decision to publish, or preparation of the manuscript. The specific roles of these authors are articulated in the ‘author contributions’ section.”

If your commercial affiliation did play a role in your study, please state and explain this role within your updated Funding Statement.

*Answer: This work was funded by Swedish government project and salary funding for clinically oriented medical research, Regional research and development grants (Southern healthcare region, Sweden;, The Crafoord Foundation and The Royal Physiographic Society in Lund). Additionally, this study was partially funded by NeuroVive Pharmaceutical AB (Lund, Sweden). NeuroVive Pharmaceutical provided support in the form of salaries for authors [S.P., J.K.E., I.C., E.E., M.J.H]. The specific roles of these authors are articulated in the ‘author contributions’ section. The funders did not have any additional role in the study design, data collection and analysis, decision to publish, or preparation of the manuscript.*

b. Please also provide an updated Competing Interests Statement declaring this commercial affiliation along with any other relevant declarations relating to employment, consultancy, patents, products in development, or marketed products, etc.

Within your Competing Interests Statement, please confirm that this commercial affiliation does not alter your adherence to all PLOS ONE policies on sharing data and materials by including the following statement: "This does not alter our adherence to  PLOS ONE policies on sharing data and materials.” (as detailed online in our guide for authors <http://journals.plos.org/plosone/s/competing-interests>) . If this adherence statement is not accurate and  there are restrictions on sharing of data and/or materials, please state these. Please note that we cannot proceed with consideration of your article until this information has been declared.

We also note that you have a patent relating to material pertinent to this article.

In the amended statement of Competing Interests please declare this patent (with details including name and number), along with any other relevant declarations relating to employment, consultancy, patents, products in development or modified products etc.

Please confirm that this does not alter your adherence to all PLOS ONE policies on sharing data and materials, as detailed online in our guide for authors <http://journals.plos.org/plosone/s/competing-interests> by including the following statement: "This does not alter our adherence to  PLOS ONE policies on sharing data and materials.” If there are restrictions on sharing of data and/or materials, please state these. Please note that we cannot proceed with consideration of your article until this information has been declared.

 Answer: S.P., J.K.E., I.C., F.S., E.E., and M.J.H have, or have had, salary from and/or equity interest in NeuroVive Pharmaceutical AB, a company active in the field of mitochondrial medicine. S.P., J.K.E., E.E., and M.J.H have filed patent applications for the use of succinate prodrugs for treatment of lactic acidosis or drug-induced side-effects due to complex I-related impairment of mitochondrial oxidative phosphorylation (WO/2015/155238) and protected carboxylic acid-based metabolites for treatment of mitochondrial disorders (WO/2017/060400, WO/2017/060418, WO/2017/060422). This does not alter our adherence to PLOS ONE policies on sharing data and materials.

c. Please include both an updated Funding Statement and Competing Interests Statement in your cover letter. We will change the online submission form on your behalf.

Please know it is PLOS ONE policy for corresponding authors to declare, on behalf of all authors, all potential competing interests for the purposes of transparency. PLOS defines a competing interest as anything that interferes with, or could reasonably be perceived as interfering with, the full and objective presentation, peer review, editorial decision-making, or publication of research or non-research articles submitted to one of the journals. Competing interests can be financial or non-financial, professional, or personal. Competing interests can arise in relationship to an organization or another person. Please follow this link to our website for more details on competing interests: <http://journals.plos.org/plosone/s/competing-interests>

*Answer: The updated Funding Statement and Competing Interests Statement are now included in the cover letter and manuscript.*

4. We note that you have included the phrase “data not shown” in your manuscript. Unfortunately, this does not meet our data sharing requirements. PLOS does not permit references to inaccessible data. We require that authors provide all relevant data within the paper, Supporting Information files, or in an acceptable, public repository. Please add a citation to support this phrase or upload the data that corresponds with these findings to a stable repository (such as Figshare or Dryad) and provide and URLs, DOIs, or accession numbers that may be used to access these data. Or, if the data are not a core part of the research being presented in your study, we ask that you remove the phrase that refers to these data.

*Answer: Any instances of ‘unpublished data’ have now been replaced with a reference to additional supplementary figures.*

__________________________________________________________________________________________________
Reviewers' comments:

Reviewer's Responses to Questions

**Comments to the Author**

1. Is the manuscript technically sound, and do the data support the conclusions?

The manuscript must describe a technically sound piece of scientific research with data that supports the conclusions. Experiments must have been conducted rigorously, with appropriate controls, replication, and sample sizes. The conclusions must be drawn appropriately based on the data presented.

Reviewer #1: Yes

Reviewer #2: Partly

2. Has the statistical analysis been performed appropriately and rigorously?

Reviewer #1: Yes

Reviewer #2: Yes

3. Have the authors made all data underlying the findings in their manuscript fully available?

The [PLOS Data policy](http://www.plosone.org/static/policies.action#sharing) requires authors to make all data underlying the findings described in their manuscript fully available without restriction, with rare exception (please refer to the Data Availability Statement in the manuscript PDF file). The data should be provided as part of the manuscript or its supporting information, or deposited to a public repository. For example, in addition to summary statistics, the data points behind means, medians and variance measures should be available. If there are restrictions on publicly sharing data—e.g. participant privacy or use of data from a third party—those must be specified.

Reviewer #1: Yes

Reviewer #2: Yes

4. Is the manuscript presented in an intelligible fashion and written in standard English?

PLOS ONE does not copyedit accepted manuscripts, so the language in submitted articles must be clear, correct, and unambiguous. Any typographical or grammatical errors should be corrected at revision, so please note any specific errors here.

Reviewer #1: Yes

Reviewer #2: No

5. Review Comments to the Author

Review Comments to the Author

Please use the space provided to explain your answers to the questions above. You may also include additional comments for the author, including concerns about dual publication, research ethics, or publication ethics. (Please upload your review as an attachment if it exceeds 20,000 characters)

Reviewer #1: The manuscript entitled “Cell-permeable succinate prodrugs rescue mitochondrial respiration in cellular models of acute acetaminophen overdose” by Piel et al investigates the effects of acute acetaminophen exposure on mitochondrial function in primary human hepatocytes, HepG2 hepatocellular carcinoma cells, and platelets. The authors demonstrate that acetaminophen inhibits complex I, but not complex II function, and that treatment with a succinate prodrug (NV241) can rescue acetaminophen-induced mitochondrial dysfunction. Overall, the paper is very well written and clearly displays the negative respiratory outcomes associated with exposure to high concentrations of acetaminophen. The concept of utilizing succinate-based prodrugs to rescue pathologies associated with mitochondrial complex I dysfunction, particularly in an acute toxic exposure scenario, is an interesting area of research. There are a few minor points that should be addressed prior to publication.

1) One potential limitation in interpretation of the respiration graphs is that data are normalized to what I presume to be an initial seeding/plating density (i.e. cell number prior to treatment). Did the authors ever measure total protein or do some assessment of viability following acetaminophen and/or the mitochondrial inhibitor exposure? It would seem that the toxicity, particularly in the case of acetaminophen, could result in significant cell death from the starting number and thus influence the overall respiratory rates.

*Answer: A valid question. As the reviewer correctly pointed out, direct measurements of cell-viability were not performed in this study. However, the increase in mitochondrial respiration in response to the cell-permeable succinate prodrug NV241, as shown in Figure 1-3, indicates intact cellular metabolism and the subsequent decrease in respiration due to antimycin A shows that this response is not unspecific chemical oxygen consumption but a biological response. The pharmacological prodrug strategy presented in this study requires intracellular metabolism to cleave off the two prodrug-moieties and release the active pharmaceutical ingredient, succinate [1]. Without intracellular metabolism, the prodrug is unable to release succinate and support mitochondrial respiration. Hence, an increase in response to the cell-permeable succinate prodrug is an indirect measure of cell viability. We demonstrate no difference between APAP-intoxicated cells and controls in response to the cell-permeable succinate prodrug NV241 with similar levels of mitochondrial respiration, indicating no difference in cell viability between the two groups. Therefore, direct measurements of cell viability were not further pursued in the present study. We will consider other approaches to assess cell viability for future studies investigating cell-permeable succinate prodrugs as treatment for APAP-induced liver damage.*

2) In Figure 1, why were rotenone and NV241 added at the same time, whereas in Figures 2 and 3 NV241 was always added post-rotenone exposure? It would seem that the minimal effect of NV241 in the primary hepatocytes may be a result of the cotreatment blocking the rescue effect.

*Answer: As rightly stated by the reviewer, primary human hepatocytes received rotenone and NV241 at the same time whereas HepG2 cells and human platelets received identical additions but in sequential order. The experiments with primary human hepatocytes were performed using the Seahorse XFe96 Analyzer (Agilent technologies, Massachusetts, USA), which allows only a total of four sequential additions per sample. The number of injections per sample is dictated by the equipment. The Seahorse XFe96 Analyzer was used for primary human hepatocytes because this instrument requires less amount of sample, which inherently is a problem when working with primary cells. In contrast, the high-resolution respirometer Oroboros-O2k (O2k, Oroboros Instruments, Innsbruck, Austria), used for the experiments with HepG2 cells and human platelets, where cell number is not a limiting factor, allows a nearly unlimited number of additions per sample. Therefore, it was possible to add rotenone and NV241 sequentially. While the mitochondrial respiration dependent on the electron transport system alone, measured following the addition of FCCP, was significantly different between APAP-intoxicated cells and controls (Fig. 1c), the respiration decreased down to the same level following the simultaneous addition of rotenone and NV241 (Fig. 1d). Hence, the simultaneous addition of rotenone with NV241 does not block the rescue effect of the cell-permeable succinate prodrug. Instead, the data show that the complex II-linked mitochondrial respiration supported by the cell-permeable succinate prodrug in the presence of rotenone (Fig. 1d) is unaffected by APAP. Therefore, for comparison of the treatment effect of NV241 between the different cell types not the response to the NV241 addition in itself should be evaluated. Instead the resulting/remaining CII-linked mitochondrial respiration in the presence of NV241 should be compared between APAP-intoxicated cells and controls. We have now clarified the corresponding results section of the manuscript.*

3) It would seem that the reliance on oxidative phosphorylation might differ significantly between a primary hepatocyte, a liver cancer cell, and a circulating platelet. Did the authors take into account ECAR/glycolysis measurements in this or previous publications to account for changes in glycolytic flux? This could be covered in the discussion.

*Answer: This is a good question, and the translatability between human platelets and tissue specific cell lines is something we continuously reevaluate. Platelets are an easily accessible and rich source of viable human mitochondria. They have previously been described to depend primarily on oxidative phosphorylation and have been regularly used for evaluation of mitochondrial function in disease and drug intoxication [2-5]. Also cancer cells, such as HepG2 cells, long believed to rely solely on glycolysis to meet their energy demand, have now been described to upregulate their mitochondrial metabolism under certain conditions and rely on mitochondrial function for several cancerogenic processes [6, 7]. Despite the difference in the absolute rate of oxidative phosphorylation the different cell types they showed similar relative sensitivity to APAP-induced mitochondrial toxicity: primary hepatocytes IC_50_ = 6.0 mM, HepG2 cells IC_50_ = 6.6 mM and human platelets IC_50_ = 7.4 mM (Fig. 4).* *The liver specific toxicity seen in patients with acute APAP intoxication is therefore likely due to the first-pass metabolism of APAP and not related to liver specific metabolism of the drug. This has now been addressed in the discussion.*

4) In Figure 2B, it appears that two of the chosen wells are accounting for the significantly higher respiration in control HepG2 cells compared to the acetaminophen treated cells (even with the indicated significant). The authors should consider removing outliers or adding in additional wells to ensure the significant decrease in respiration that occurs following acetaminophen exposure in these cells.

*Answer: We appreciate the attention to detail. Like the reviewer excellently spotted there were two replicates in Figure 2B which showed higher respiration than the remaining replicates of the experiment. We now have performed an outlier identification analysis. ROUT outlier analysis with a maximally desired false discovery rate of 1% detected no outliers in either the control or APAP group. Further data analysis showed that the difference among the six replicates is independent of APAP, as illustrated below. The two replicates which showed a higher routine respiration following exposure to vehicle or APAP also showed a higher routine respiration before start of exposure. Further, a high respiration of vehicle-treated cells matched a high respiration of the APAP-intoxicated cells of the same replicate, as now indicated by individual color coding of each replicate in the below figure.*

5) In Figure 5A, why does the FCCP titration taper off in the acetaminophen treated cells and then recover to control following rotenone treatment? Was this a result of negative readings/values? The authors could discuss in the results or discussion section.

*Answer: We agree with the reviewer that the illustration in Figure 5A is misleading regarding the response to FCCP. The decrease in mitochondrial respiration following the last addition of FCCP in the APAP-treated cells is not a response to the protonophore FCCP but an artifact due to the injection in itself. The Oroboros O2k measures cellular respiration by calculating the negative derivative slope of the oxygen concentration in your sample. By injecting solutions to the sample, like in the described SUIT protocol, oxygen is simultaneously injected. This creates an artificial increase in oxygen concentration in the sample and hence, is interpreted by the software as a temporary decrease in respiration. This event is independent of the respiratory rate of the sample. Once the oxygen, that is injected with the solution, is equally distributed in the sample, the respiration signal is reflecting only the cellular respiration of the sample again. We agree with the reviewer that the selection where the mitochondrial respiration trace of the APAP-intoxicated sample was cut and adjusted to allow for comparison of the representative traces of both groups might be misleading. We have improved Figure 5A accordingly and included a clarification in the corresponding figure legend.*

Reviewer #2: While the manuscript submitted uses a variety of respirometry methods to accurately assess mitochondrial respiration (and therefore metabolism), as written, the manuscript makes conclusions that are "reaching" and not entirely supported by the data. Below are the major concerns that are outlined - these revision experiments would make the manuscript suitable for publication.

Major Concerns:

The authors present their succinate prodrug as an adjunctive therapy to NAC, yet do not do a single experiment comparing their prodrug to NAC, nor any combination therapy experiments. If the conclusion that this prodrug is adjunctive, they must show that in their experimental models.

*Answer: A valid concern. The mechanism of action of N-acetylcysteine (NAC) and the novel, mitochondrial-targeted therapy of cell-permeable succinate prodrugs are very different. NAC protects liver cells from further oxidative damage by APAP by increasing glutathione levels and replenishing the cell’s antioxidant defense [8-10]. The cell-permeable succinate prodrug, in contrast, aims at rescuing already damaged cells by increasing mitochondrial respiration which is coupled to phosphorylation pathways and thereby potentially rescues the otherwise dying cells. Because both treatment strategies have a different mechanism of action and target different populations of liver cells in the situation of acute APAP intoxication we do think their treatment effects cannot be compared using the here described methodology: respirometry. We do agree with the reviewer that therefore our conclusions that the cell-permeable succinate prodrugs could be adjunctive therapies are too far reaching and have consequently removed these statements from the manuscript.*

The authors did not assess the effect of their prodrug on any endpoints apart from mitochondrial respiration. While this reviewer appreciates the rationale (that this prodrug is specifically rescuing mitochondrial respiration), it would be interesting to see how this drug impacts downstream endpoints of cellular health that are affected by acetaminophen toxicity, such as ROS production, apoptosis/necrosis, cell proliferation, etc.

Pursuant to the previous comments, assessing downstream cell health endpoints after combination therapy would greatly strengthen the authors conclusions.

*Answer: The suggestions by the reviewers are all very valid points, and we agree that the study could be further expanded this way. The compound NV241 is a first-of-its-kind succinate prodrug, previously reported as an investigative compound for primary mitochondrial disease [1]. It is a model drug of a first generation optimized for relatively short-term in vitro experiments, with very short half-life in plasma (minutes in humans, seconds in rodents). This makes the possible experimental designs somewhat limited. Oxidative damage and cellular death are cellular endpoints that have been reported to appear not earlier than 3-6 h following APAP ingestion and therefore would require drug candidates which demonstrate a longer stability [10]. Newer generations of compounds in the same pharmaceutical class, with better stability, are under investigation and will be applied to this project when possible. This is a clear limitation of this study and we have included a section on limitations of this study in the discussion part to reflect the lack of the above-mentioned models.*

Minor Concerns:

The manuscript is generally well-written, however it would benefit from a thorough edit for syntax, use of commas, plurals, etc.

*Answer: The reviewer is correct, there was room for improvement regarding the language quality. We have gone through the manuscript to further improve the language.*

1.	Ehinger, J.K., et al., *Cell-permeable succinate prodrugs bypass mitochondrial complex I deficiency.* Nat Commun, 2016. **7**: p. 12317.

2.	Kramer, P.A., et al., *Bioenergetics and the oxidative burst: protocols for the isolation and evaluation of human leukocytes and platelets.* J Vis Exp, 2014(85).

3.	Kramer, P.A., et al., *A review of the mitochondrial and glycolytic metabolism in human platelets and leukocytes: implications for their use as bioenergetic biomarkers.* Redox Biol, 2014. **2**: p. 206-10.

4.	Ravi, S., et al., *Mitochondria in monocytes and macrophages-implications for translational and basic research.* Int J Biochem Cell Biol, 2014. **53**: p. 202-207.

5.	Garcia-Souza, L.F. and M.F. Oliveira, *Mitochondria: biological roles in platelet physiology and pathology.* Int J Biochem Cell Biol, 2014. **50**: p. 156-60.

6.	Shiratori, R., et al., *Glycolytic suppression dramatically changes the intracellular metabolic profile of multiple cancer cell lines in a mitochondrial metabolism-dependent manner.* Scientific Reports, 2019. **9**(1): p. 18699.

7.	Porporato, P.E., et al., *Mitochondrial metabolism and cancer.* Cell Research, 2018. **28**(3): p. 265-280.

8.	Saito, C., C. Zwingmann, and H. Jaeschke, *Novel mechanisms of protection against acetaminophen hepatotoxicity in mice by glutathione and N-acetylcysteine.* Hepatology, 2010. **51**(1): p. 246-54.

9.	Lee, K.K., et al., *Targeting mitochondria with methylene blue protects mice against acetaminophen-induced liver injury.* Hepatology, 2015. **61**(1): p. 326-36.

10.	Hinson, J.A., D.W. Roberts, and L.P. James, *Mechanisms of acetaminophen-induced liver necrosis.* Handb Exp Pharmacol, 2010(196): p. 369-405.
